# *In vitro* digestion and fermentation of ginseng pectic polysaccharide GPS-1 and attenuation of its product on oleic acid-induced oxidative stress in HepG2 cells 

**DOI:** 10.3389/fphar.2026.1758770

**Published:** 2026-02-18

**Authors:** Xiwen Sun, Li Liu, Wenbo Jiao, Ting Ren, Ran Zhao, Zirui Tan, Ziye Jiang, Jing Wang, Bo Li, Xiaoyu Zhang, Lili Jiao

**Affiliations:** 1 Jilin Ginseng Academy, Changchun University of Chinese Medicine, Changchun, China; 2 The Affiliated Hospital Changchun University of Chinese Medicine, Changchun University of Chinese Medicine, Changchun, China; 3 School of Pharmaceutical Sciences, Jilin Medical University, Jilin, China; 4 School of Pharmaceutical Sciences, Changchun University of Chinese Medicine, Changchun, China; 5 The Third Affiliated Hospital of Changchun University of Chinese Medicine, Changchun University of Chinese Medicine, Changchun, China

**Keywords:** ginseng, HepG2 cells, in vitro digestion and fermentation, oxidative stress, polysaccharide

## Abstract

**Background:**

Based on prior evidence that red ginseng pectin GPS-1 ameliorates T2DM in rats and modulates gut microbiota, we report for the first time that GPS-1 interacts with the gut microbiota of T2DM rats, as demonstrated through an *in vitro* digestion-fermentation model. This study reports for the first time that GPS-1 is key to its anti-T2DM efficacy. Building on prior findings that GPS-1 modulates gut microbiota in diabetic rats, we employed an *in vitro* digestion-fermentation model to demonstrate how GPS-1 is metabolized by specific bacteria into beneficial metabolites (e.g., short-chain fatty acids), thereby clarifying the causal pathway through which GPS-1 improves host metabolic health.

**Materials and methods:**

GPS-1 was subjected to simulated salivary-gastrointestinal digestion *in vitro*, followed by fecal fermentation. Its physicochemical properties, including molecular weight (*Mw*), monosaccharide composition, uronic acid and reducing sugar content, were monitored. Short-chain fatty acids (SCFAs) production was quantified by GC-MS, and changes in gut microbiota composition were analyzed by 16S rRNA sequencing. The hepatoprotective effect of the fermented product (GPS-1-I48) was evaluated in oleic acid-treated HepG2 cells by measuring levels of triglyceride (TG), malondialdehyde (MDA), and the activities of superoxide dismutase (SOD), catalase (CAT), and glutathione peroxidase (GSH-Px).

**Results:**

GPS-1 was highly resistant to *in vitro* digestion, with no significant changes in *Mw* or carbohydrate composition. However, it was effectively degraded during fermentation, showing marked decreases in *Mw* and uronic acid content, a shift in monosaccharide profile, and an increase in reducing sugars. Fermentation of GPS-1 significantly modulated the gut microbiota structure of T2DM rats. It also markedly promoted the production of SCFAs. Consequently, the fermented product GPS-1-I48 exhibited significantly enhanced hepatoprotective activity, increasing SOD, CAT, and GSH-Px activities while reducing MDA and TG levels in HepG2 cells.

**Conclusion:**

This study demonstrates that the hepatoprotective effect of GPS-1 depends on gut microbial fermentation. GPS-1 resists digestion but is degraded by the microbiota, enriching *Bacteroides*, boosting propionate and acetate production, and generating fermented products with enhanced antioxidant and lipid-lowering activity. These findings suggest that GPS-1 has potential as a prebiotic functional ingredient for improving intestinal health and regulating lipid metabolism.

## 1 Introduction

The intestine serves as the most vital digestive and immune organ in humans (Dilip et al., 2019), hosting a vast and dynamic microbial ecosystem known as the gut microbiota. The gut microbiota performs essential physiological functions including metabolism, nutrient assimilation, and host protection, thereby profoundly influencing human health (Gilbert et al., 2018). Conversely, its structural dysbiosis has been closely associated with the pathogenesis of various diseases such as metabolic syndrome, diabetes, obesity, inflammatory bowel disease, and malignancies (Ding et al., 2020; Schlegel et al., 2019). In this context, the bidirectional interaction between polysaccharides and the gut microbiota has attracted increased attention. On one hand, the gut microbiota functions as a key agent in polysaccharide breakdown through the ability of its constituent microorganisms, especially *Bacteroides* species, to encode numerous carbohydrate-active enzymes (CAZymes) and possess abundant polysaccharide utilization loci (PULs) (Wardman et al., 2022). This enzymatic machinery enables the degradation of indigestible plant polysaccharides into monosaccharides or oligosaccharides via specialized degradation and transport systems upon their arrival in the colon. These resulting products are then internalized to participate in host metabolic regulation (Lv et al., 2018). On the other hand, the microbiota is in turn modulated by plant polysaccharides. As an important dietary component, these polysaccharides operate by selectively promoting beneficial bacterial growth and inhibiting potential pathogens. This process reshapes gut microbial structure, improves intestinal homeostasis, and ultimately exerts health-modulating effects.

Beyond serving as substrates for microbial degradation, polysaccharides undergo sequential fermentation through cascade reactions, enabling efficient production of bioactive compounds such as short-chain fatty acids (SCFAs) (Campos-Perez and Martinez-Lopez, 2021). These metabolites play crucial roles in protecting intestinal epithelial cells, preventing colorectal carcinogenesis, reducing intestinal pH, inhibiting pathogenic bacterial proliferation, and enhancing intestinal mucosal immunity (Kim et al., 2014). Meanwhile, as carbon sources, polysaccharides promote the growth of beneficial bacteria, thereby improving intestinal integrity, alleviating mucosal damage, modulating microbial composition, and upregulating functional enzyme activities (Elshahed et al., 2021). These multidimensional effects collectively regulate gut microbiota at structural, compositional, and functional levels. Therefore, current research suggests that microbial degradation of polysaccharides constitutes the fundamental mechanism through which these compounds exert their pharmacological effects.

Pectin mainly composed of homogalacturonan (HG), rhamnogalacturonan I (RG-I) and rhamnogalacturonan II doamins (RG-II), Arabinogalactan (AG), and Xylogalacturonan (XG), and has good biological activity and low toxicity (Mohnen et al., 2008). Pectin has been shown to be a potentially active substance in the treatment of diabetes mellitus. They showed antidiabetic effects directly or indirectly by modulating β-cell function (Wang et al., 2016), regulating sugar and lipid metabolism (Li et al., 2021), inhibiting α-amylase and α-glucosidase activity (Hamden et al., 2018), inhibiting oxidative stress (Sun et al., 2014), anti-inflammation (Esser et al., 2014), and immunomodulation (Wu et al., 2021b). Importantly, as a complex polysaccharide, pectin is difficult to be digested by the organism, but can be degraded and utilized by enzymes encoded by intestinal microflora, ultimately producing SCFAs, including acetic, propionic and butyric acids, etc. (Campos-Perez and Martinez-Lopez, 2021). SCFAs are important active substance in the intestinal that participated in regulating lipid metabolism by binding to G-protein-coupled receptors (GPR41/GPR43). Acetic acid as one of the key substrate in biosynthesis of cholesterol and fatty acid, and can reduce free fatty acid levels (Kimura et al., 2019); Propionic acid, as a precursor of glucose synthesis in the liver, can lower cholesterol content via inhibiting Acetyl-CoA synthetase activity (Qin et al., 2020); Butyric acid may prevent diet-induced obesity, hepatic steatosis and insulin resistance by improving energy metabolism and mitochondrial function (Mandaliya and Seshadri, 2019). Pections from various plants have been found to increase the species and abundance of SCFAs producing bacteria by modulating the gut microbiota, thereby improving the glycolipid metabolism of T2DM rats (Hamden et al., 2018; Wu et al., 2017).

Interestingly, pectin can be degraded by gut microbiota, but also serve as a carbon source to promote the growth of intestinal bacteria, regulate the intestinal microbiota structure and its metabolites, thereby improving the integrity of the intestinal function, alleviating intestinal mucosal injury, and ultimately promoting gut health (Ren et al., 2025). Members of the *Bacteroidetes* phylum and *Prevotella* genus harbor abundant genes encoding CAZymes. These microorganisms employ pectin lyases, methyl esterases, and acetyl esterases to degrade pectin molecules. The degraded pectin derivatives are subsequently metabolized by gut bacteria, modulating microbial community structure to enhance systemic metabolism. This mechanism provides a potential therapeutic strategy for diabetes management (Martens et al., 2011). Lee et al. found that pectin from *Corchorus olitorius* L. leaves can improve blood glucose and insulin levels, and reduce the Firmicutes/Bacteroidetes (F/B) values of gut microbiota in overweight patients (Lee et al., 2022). Pianta et al. and Pedersen et al. found that low methoxylated citrus pectin has been found to reduce insulin resistance by increasing the abundance of *Prevotella* (Pianta et al., 2017; Pedersen et al., 2016).

*Panax ginseng* C.A.Mey. (*P. ginseng*) is one of the important food-medicine herbs which contains a variety of active ingredients such as ginseng saponins, polysaccharides, and has good effects in lowering blood glucose, improving lipid metabolism disorders, and enhancing insulin sensitivity (Ziaei et al., 2020; Hasanpour et al., 2020). Polysaccharides are the major components in ginseng and have been found to have hypoglycaemic and hypolipidemic effects (Zhao et al., 2019). Our previous study found that the pectin-like polysaccharides (GPS-1) form the red ginseng (steamed at 120 °C) significantly reduced the lipid accumulation in the liver of T2DM rats through regulating the gut microbiota (Ren et al., 2023). However, the digestive and fermentation characteristics of GPS-1 are still unclear.

Therefore, in this study, we will employ an *in vitro* simulated digestion and fermentation model to investigate the changes in physicochemical properties and structural features of GPS-1, as well as its impacts on intestinal microbiota structure and SCFA content. Furthermore, we will evaluate how the digestion and fermentation processes modulate the effects of GPS-1 in improving OA-induced damage in HepG2 cells. Our findings will provide useful information for understanding the digestive and fermentative behaviors of GPS-1 and provide theoretical basis for the application of GPS-1 as a potential probiotic to improve intestinal health.

## 2 Materials and methods

### 2.1 Experimental materials

*P. ginseng* (5-year-old) was cultivated in Tonghua City, Jilin Province, China. Dulbecco′s Modified Eagle′s Medium (DMEM, catalog no. C11330500BT, Gibco/Thermo Fisher Scientific) and Fetal Bovine Serum (FBS, Cat. No. A5256701, Gibco/Thermo Fisher Scientific) were used. Catalase (CAT, catalog no. A007-1-1), superoxide dismutase (SOD, catalog no. A001-3-1), glutathione peroxidase (GSH-Px, catalog no. A005-1–2), malondialdehyde (MDA, catalog no. A003-4-1), triglyceride (TG, catalog no. A110-1-1) and kits were purchased from Nanjing Jiancheng Bioengineering Institute (Nanjing, China). Cell Counting Kit-8 (CCK-8, catalog no. BS350B) were purchased from Beijing abgic Technology Co., Ltd. All additional chemicals and reagents were of analytical reagent grade.

### 2.2 Experimental method

#### 2.2.1 Preparation of the GPS-1

The polysaccharide (GPS-1) was isolated from red ginseng according to a previously method reported (Ren et al., 2023). Briefly, GPS-1 was extracted from steaming ginseng using hot water extraction and ethanol precipitation. Proteins were removed using Sevag reagent, and the resulting products was sequentially purified through a DEAE-cellulose column and a Sepharose CL-6B gel column. The obtained and purified GPS-1 was characterized as a homogeneous polysaccharide by high-performance gel permeation chromatography (HPGPC), showing an average molecular weight (*Mw*) of approximately 1.237 × 10^5^ Da (Supplementary Figure S1). Its protein content was determined to be less than 0.5% using the Bradford assay (Yang et al., 2023). To rule out significant endotoxin contamination that could interfere with subsequent bioassays, we also measured the endotoxin level in GPS-1 with the ToxinSensor™ Chromogenic LAL Endotoxin Detection Kit (Li et al., 2025). The result confirmed that the endotoxin content was below the detection limit of 0.01 EU/mL.

#### 2.2.2 General methods

The total sugar content of the samples was determined using phenol-sulfuric acid method (Dubois et al., 1951), the glucuronic acid content was determined using the m-hydroxybiphenyl method with galacturonic acid (GalA) as the standard (Blumenkrantz and Asboe-Hansen, 1973). The Coomassie Brilliant Blue assay was used to determine the protein content using bovine serum albumin (BSA) as the standard (Grintzalis et al., 2015). The *Mw* was determined by HPGPC employing standard dextrans to draw the standard curve. The analysis was conducted on a Shimadzu HPLC system equipped with a TSKgel G3000 PWxL column (7.8 mm × 30.0 cm) and an RID-20A detector. A 0.2 M NaCl mobile phase was delivered at 0.5 mL/min with the column temperature maintained at 40 °C. The injection volume was 10 μL. 3, 5-dinitrosalicylic acid (DNS) method was used to determine the content of reducing sugar (C_R_) (Wu et al., 2021a). The values of pH and optical density at 600 nm (OD_600_) of the fermentation broth at different time points were determined using pH Meter (Mettler Toledo, Switzerland) and microplate reader (Infinite M200 Pro, Switzerland), respectively.

#### 2.2.3 Analysis of monosaccharide composition

The monosaccharide composition of the samples was determined via PMP-pre-column derivatization combined with HPLC analysis (Yang et al., 2023). 3 mg of the polysaccharide was first hydrolyzed with 2 M HCl in methanol and then 2 mol/L trifluoroacetic acid (TFA), subsequently. The hydrolysate was derivatized with 0.5 mL of PMP reagent. After filtration, the PMP derivatives were analysed on a HPLC system (UlitiMate 3,000 instrument, ThermoFinningan, United States).

#### 2.2.4 FT-IR analysis

The sample (2 mg) was dried 24 h and mixed with 200 mg KBr thoroughly. The resulting mixture was made into KBr tablets and the FT-IR spectra were measures using a Thermo Scientific Nicolet iS5 FT-IR spectrometer (Thermo Fisher Scientific, United States), within the wave number range of 4,000 to 400 cm^-1^.

#### 2.2.5 *In vitro* saliva-gastric-interstinal digestion of GPS-1

The artificial simulated digestion was conducted according to the established protocols (Xu et al., 2025; Guo et al., 2022). Briefly, 383.0 mg of NaCl, 745.4 mg of KCl, 66.5 mg of CaCl_2_, and 500 mg of α-amylase were dissolved in 500 mL of deionized water, and the pH was adjusted to 6.8 with 1 mol/L HCl and/or 1 mol/L NaHCO_3_. Subsequently, 250 mL of GPS-1 (10 mg/mL) was mixed with an equal volume of the simulated saliva and incubated at 37 °C in a water bath shaker. Digestive samples were collected at 10 min and 20 min during the process.

The remaining saliva-digested GPS-1 sample was mixed with an equal volume of simulated gastric juice. The gastric electrolyte solution (GES) was prepared by dissolving 550.0 mg of NaCl, 550.0 mg of KCl, 75.0 mg of CaCl_2_⋅2H_2_O, and 150.0 mg of NaHCO_3_ in 500 mL deionized water, followed by adjusting the pH to 3.0 with 0.1 M HCl. To the mixture, 112.5 mg of gastric lipase, 106.2 mg of pepsin, and 4.5 mL of CH_3_COONa solution (1 M, pH 5.0) were added, and the final pH was adjusted to 2.0. Aliquots (20 mL) of the gastric digestate were collected after incubation at 37 °C for 0.5, 1, 2, and 4 h.

The remaining gastric-digested GPS-1 sample was mixed with an equal volume of simulated intestinal juice (composition: 92.4 mmol/L NaCl, 8.72 mmol/L KCl, 2.25 mmol/L CaCl_2_·2H_2_O, 17.5 mg/mL pancreatin, 20 mg/mL bile salt in 450 mL deionized water) and the pH was adjusted to 7.0. After incubation at 37 °C for 0.5, 1, 2, and 4 h, 20 mL aliquots of the intestinal digestate were collected.

The digestion products from different phase (GPS-1-S: saliva digestion; GPS-1-G: gastric digestion; GPS-1-I: intestinal digestion) were recovered by ethanol precipitation.

#### 2.2.6 *In vitro* fermentation of GPS-1 (GPS-1-I)

The fecal microbiota was prepared according to the previous reported method (Xu et al., 2025). Briefly, fresh fecal samples were collected from ten T2DM rats which was fed with HG/HF diet (for 8 weeks) and injected with 35 mg/kg of STZ (Ren et al., 2023). The animal study was performed in compliance with the Guidelines for the Care and Use of Laboratory Animals and was approved by the Institutional Animal Care and Use Committee of Changchun University of Chinese Medicine (Approval No.: 2021055). The feces (5 g) was mixed with PBS (0.1 M, pH = 6.8) at a concentration of 10% (w/v), centrifuged and the fecal suspension was collected for further use. The basic nutrient medium (1 L) contained the following components: 2.0 g peptone, 2.0 g yeast extract, 0.5 g cysteine-HCl, 0.1 g NaCl, 2.0 g NaHCO_3_, 0.04 g K_2_HPO_4_, 0.04 g KH_2_PO_4_, 0.01 g MgSO_4_7H_2_O, 0.01 g CaCl_2_ 6H_2_O, 0.02 g heme, 0.5 g bile salt, 2.0 mL Tween 80, 10 μL vitamin K1, and 1.0 mL resazurin (1% w/v).

The fecal fermentation system was prepared by mixing 50 mL of fecal supernatant with 450 mL of the basic nutrient medium. For GPS-1 group, 5.0 g of GPS-1 was added to this system. A control group, containing only the basic nutrient medium and fecal supernatant, was also prepared. Both groups were then cultured in an anaerobic condition at 37 °C. The fermented samples (20 mL) at different time points (12, 24, 36, and 48 h) were collected for further use. One part of the fermented sample was used to detect the values of pH and OD_600_, the other part was precipitated with 95% ethanol to obtain the polysaccharides: GPS-1-I12, GPS-1-I24, GPS-1-I36 and GPS-1-I48.

#### 2.2.7 Gut microbiota analysis

After 48 h of fermentation, the sample was centrifuged to collect the sediment for DNA extraction. The V3-V4 hypervariable regions of 16S rRNA were amplified and sequenced on the Illumina NovaSeq platform (Biomarker Technologies, Beijing, China). All data analysis were performed using BMKCloud (www.biocloud.net).

#### 2.2.8 SCFAs content analysis

A gas chromatograph-mass spectrometer (TSQ8000; Thermo Fisher Scientific, United States) equipped with a TR-FFAP column (30 m × 0.25 mm × 0.25 μm) was used to quantify SCFAs. The injection volume was 1 μL. The chromatogram temperature program was set as follows: initial hold at 100 °C for 1 min, ramped to 150 °C at 5 °C/min and held for 5 min, then further increased to 200 °C at 25 °C/min and maintained for 2 min.

#### 2.2.9 Effects of GPS-1 and its digestion products on oleic acid-induced HepG2 cells

##### 2.2.9.1 Cell culture

HepG2 cells were cultured in complete DMEM medium containing 10% fetal bovine serum, 100 U/mL penicillin and 100 μg/mL streptomycin, and maintained in a humidified incubator at 37 °C with 5% CO_2_.

##### 2.2.9.2 Cytotoxicity assay

HepG2 cells were seeded in 96-well cell plates at a density of 2.5 × 10^5^ cells/mL (100 μL per well). Cell viability was evaluated via the CCK8 assay after treatment with various sample concentrations (50, 100, 200, 400, and 800 μg/mL). Absorbance at 450 nm was measured using a microplate reader, and cell survival rates were calculated relative to untreated controls.

##### 2.2.9.3 Examination of the effects of GPS-1 on OA-induced HepG2 cells

The lipid metabolism disorder cell model was established according to the method reported by He et al. (2018), and made some modifications. Briefly, the cells were seeded in a 96-well culture plate (2.5 × 10^4^ cells/well), cultured in serum-free medium for 12 h and then treated with 200 μM oleic acid to induce lipid accumulation. Subsequently, the cells were incubated with samples (GPS-1 and GPS-1-I48 at 100,200 and 400 μg/mL) for 24 h at 37 °C. Cell viability was evaluated via the CCK8 assay, and cell survival rates were calculated relative to untreated controls.

##### 2.2.9.4 Oil Red O staining

The HepG2 cells were seeded in a 12-well culture plate (3 × 10^5^ cells/well), cultured in serum-free medium for 12 h and then treated with 200 μM oleic acid to induce lipid accumulation. Subsequently, the cells were incubated with samples (GPS-1 and GPS-1-I48 at 100, 200 and 400 μg/mL) for 24 h at 37 °C. Following culture termination, the medium was aspirated and the cells were washed with PBS for two times. After rinsing with 60% isopropanol, lipid deposition was stained with Oil Red O solution (15–20 min), followed by nuclear counterstaining with Mayer’s hematoxylin and observation using an optical microscope. Subsequently, after the cells were thoroughly washed, carefully aspirate the supernatant. Then, add 500 μL of anhydrous isopropanol to each well, mix thoroughly by shaking, and incubate for 10 min. Afterwards, transfer the solution containing the dissolved dye to a new 96-well plate. Measure the absorbance at a wavelength of 510 nm using a microplate reader.

##### 2.2.9.5 Analysis of SOD, CAT, GSH-Px activities, MDA and TG levels

OA-induced HepG2 cells were treated with GPS-1 and GPS-1-I48 (100–400 μg/mL) for 24 h, and the supernatants were collected to detect the activities of SOD, CAT, GSH-Px, and MDA level using commercial assay kits (Nanjing Jiancheng Bioengineering Institute, China). TG content was measured in accordance with the manufacturer’s protocol (Nanjing Jiancheng Bioengineering Institute, China).

#### 2.2.10 Statistical analysis

All experiments were performed in three repetitions. The dates were analyzed using GraphPad Prism 6 software and expressed as mean ± S.D. The differences between groups were analyzed using the one-way ANOVA method, and *P* < 0.05 was considered a significant different.

## 3 Results

### 3.1 Dynamic changes in total sugar content, uronic acid, and C_R_ levels of GPS-1 during digestion and fermentation

As shown in Table 1, the total sugar content of GPS-1 decreased from 17.27 mg/mL to 15.80 mg/mL during stimulated salivary, gastric, and small intestinal digestion. Notably, during fermentation with T2DM fecal microbiota, the total sugar content was further reduced to 3.76 mg/mL, corresponding to a consumption rate of 76.20%. In addition, C_R_ levels of GPS-1 remained stable throughout most digestion process. A minor but significant increase was during simulated gastric digestion (0.0518 ± 0.0022 mg/mL to 0.0561 ± 0.0017 mg/mL), suggesting partial degradation of non-amyloid polysaccharides under acid gastric conditions with digestive enzymes. During the fermentation, C_R_ concentration increased slowly from 0–12 h, followed by rapid elevation from 12–24 h (0.0793 ± 0.0016 mg/mL to 0.1387 ± 0.0099 mg/mL). Interestingly, C_R_ levels progressively decreased during the end stage of fermentation (36–48 h). The dynamic changes in uronic acid content during digestion and fermentation are shown in Table 2. During simulated digestion (saliva, gastric and small intestinal phases), the uronic acid content decreased from 18.168 mg/mL to 16.011 mg/mL. Notably, a marked reduction occurred in the fermentation process, with uronic acid content decreasing from 10.563 mg/mL to 6.677 mg/mL between 12 h and 24 h. These results indicate that GPS-1 was predominantly degraded by gut microbiota, with accelerated fermentative activity observed during the 12–24 h fermentation.

**TABLE 1:** Changes in total and C_R_ content of GPS-1 during digestion and fermentation *in vitro*.

Processes	Time (h)	Total sugar (mg/mL)	C_R_ (mg/mL)
Saliva digestion	0	17.27 ± 0.13	0.0502 ± 0.0010
	0.17	17.27 ± 0.12	0.0501 ± 0.0009
	0.33	17.24 ± 0.17	0.0518 ± 0.0022
Gastric juice digestion	0.5	17.03 ± 0.47	0.0515 ± 0.0013
	1	17.00 ± 0.05	0.0525 ± 0.0006
	2	16.97 ± 0.20	0.0544 ± 0.0032
	4	16.64 ± 0.09	0.0561 ± 0.0017
Small intestinal juice digestion	0.5	16.23 ± 2.82	0.0566 ± 0.0918
	1	15.03 ± 3.66	0.0565 ± 0.0029
	2	15.94 ± 1.01	0.0563 ± 0.0010
	4	15.80 ± 2.11	0.0569 ± 0.0015
*In vitro* fecal fermentation	12	13.35 ± 0.85^*^	0.0793 ± 0.0016
	24	7.24 ± 0.41^**^	0.1387 ± 0.0099^**^
	36	4.75 ± 0.19^**^	0.1373 ± 0.0045^**^
	48	3.76 ± 0.171^**^	0.095 ± 0.0059^*^

^*^ *P* < 0.05, ^**^ *P* < 0.01 vs. 0-h oral digestion.

**TABLE 2:** Changes in uronic acid and *Mw* during digestion and fermentation of GPS-1 *in vitro*.

Samples	Uronic acid (mg/mL)	*Mw* (Da)
GPS-1	18.168 ± 0.080	1.237 × 10^5^
GPS-1-S	18.248 ± 0.193	1.236 × 10^5^
GPS-1-G	17.738 ± 0.167	1.183 × 10^5^
GPS-1-I	16.011 ± 0.156	1.178 × 10^5^
GPS-1- I12h	10.563 ± 0.206^**^	1.065 × 10^5^
GPS-1- I24h	6.677 ± 0.145^**^	5.012 × 10^4^
GPS-1- I36h	5.957 ± 0.032^**^	1.343 × 10^4^
GPS-1- I48h	5.330 ± 0.466^**^	4.168 × 10^3^

^*^ *P* < 0.05, ^**^ *P* < 0.01 vs. GPS-1.

### 3.2 Changes in *Mw*, monosaccharide composition, FT-IR spectra and degree of esterification of GPS-1 during digestion

**FIGURE 1:**
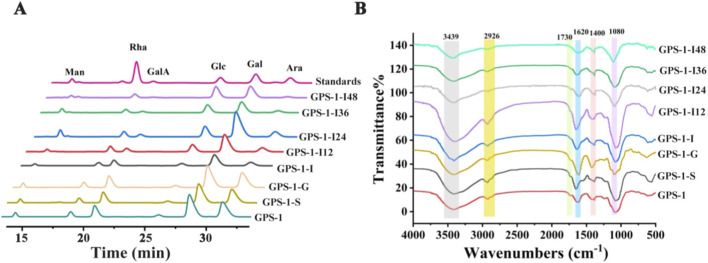
Changes in the monosaccharide composition **(A)** and FT-IR **(B)** of GPS-1 during digestion and fecal fermentation *in vitro*.

In this work, we investigated the changes of *Mw*, monosaccharide composition, and functional group of GPS-1 using the *vitro* digestion and fermentation model. As shown in Table 2, the *Mw* of GPS-1 declined from 1.237 × 10^5^ Da to 1.178 × 10^5^ Da following sequential digestion by saliva, gastric juice, and small intestinal fluids, and further decreased to 4.168 × 10^3^ Da after 48 h of fecal fermentation. In addition, no significant change in monosaccharide composition was observed from oral to gastric stages, but the content of Ara changed greatly during digestion in the small intestine, decreasing from 32.94% to 25.06% (Figure 1A; Table 3). During the *in vitro* fecal fermentation process, the monosaccharide composition of GPS-1 demonstrated significant alterations at different time points. Notably, after 48 h of fermentation, the relative content of GalA declined from 14.77% to 2.06%. Conversely, Glc and Fuc showed an increase from 5.27% to 35.29% and 0.15%–2.38%, respectively. According to the FT-IR results (Figure 1B), GPS-1 and its digestive products (GPS-1-S, GPS-1-G, and GPS-1-I) and fermented products (GPS-1-I12 h, GPS-1- I24 h, GPS-1-I36 h, and GPS-1-I48 h) exhibited similar FT-IR spectral profiles. The broad absorption peak near 3,439 cm^-1^ corresponds to O-H stretching vibration, the weak absorption peak around 2,926 cm^-1^ arises from C-H stretching vibration in saccharide rings, and the absorption at 1,400 cm^-1^ can be attributed to the existence of the C-H stretching vibration in polysaccharides. The absorption peaks around 1,620 cm^-1^ reflects COO^−^ asymmetric stretching vibration. Notably, the esterified carboxylic group C=O stretching vibration at 1730 cm^-1^ maintained stability during digestive phase but exhibited progressive intensity attenuation with the increase in fermentation time. The decrease of esterification correlated with the observed uronic acid content dynamics.

**TABLE 3:** Changes in the monosaccharide composition of GPS-1 during digestion and fecal fermentation *in vitro*.

Monodaccharide (mol%)	GPS-1	GPS-1-G	GPS-1-S	GPS-I-0h	GPS-I-12h	GPS-I-24h	GPS-I-36h	GPS-I-48h
Man	3.43	3.52	3.63	4.06	3.73	6.51	7.93	4.10
GlcA	0.26	0.20	0.20	0.41	0.36	0.32	0.34	0.57
Rha	7.88	7.11	7.12	7.29	7.84	8.41	9.17	9.71
GalA	12.61	14.63	14.41	14.77	6.60	3.54	3.79	2.06
Glc	4.71	4.68	4.88	5.27	16.54	17.26	26.55	35.29
Gal	37.81	35.83	36.64	42.63	49.22	50.95	40.63	40.71
Xyl	0.11	0.12	0.11	0.36	0.50	0.55	0.64	0.85
Ara	33.13	33.84	32.94	25.06	14.55	11.17	9.11	4.33
Fuc	0.06	0.07	0.07	0.15	0.66	1.29	1.84	2.38

### 3.3 Changes in OD_600_ and pH

**FIGURE 2:**
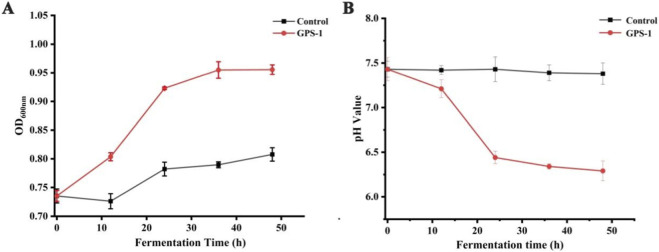
Effect of GPS-1 on OD600 nm **(A)** and pH **(B)** of fecal digests of T2DM rats.

The OD_600_ serves as a reliable indicator of the bacterial biomass in the fermentation broth. As shown in Figure 2A, GPS-1 supplemented group exhibited significant dynamic changes in OD_600_ values during the whole fecal fermentation process. Compared with the normal group, the OD_600_ of GPS-1 fermentation broth increased rapidly from 0.74 ± 0.01 to 0.92 ± 0.00 during the initial 24-h phase, peaking at 36 h. This growth pattern indicates that GPS-1 enhances the proliferation of the microbiota of T2DM rat feces. At the same time, a decrease in pH of the fermentation broth was observed, and the PH values of GPS-1 group remained consistently lower than those of the control group at all fermentation timepoints (Figure 2B).

### 3.4 GPS-1 affected the composition of gut microbiota during *in vitro* fecal fermentation

#### 3.4.1 Diversity analysis

**FIGURE 3:**
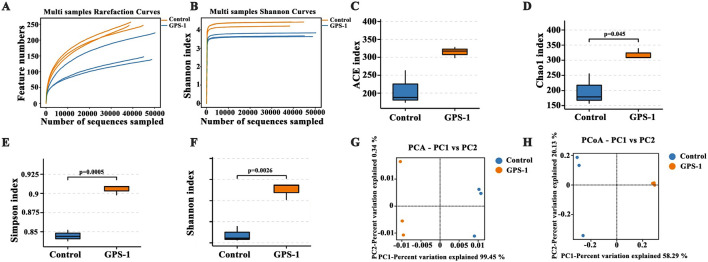
Effects of GPS-1 on gut microbiota: **(A)** Rarefaction curve; **(B)** Shannon curves; **(C)** Ace index; **(D)** Chao1 index; **(E)** Simpson index; **(F)** Shannon index; **(G)** Beta diversity (PCA); **(H)** Beta diversity (PCoA). ^*^ *P* < 0.05, ^**^ *P* < 0.01 compared with the control group.

To investigate the modulatory effects of GPS-1 on gut microbiota, we first conducted the α- and β-diversity analysis through full-length 16S rRNA sequencing. As shown in Figures 3A,B, the curve tends to flatten with the increase of the sample size and sequencing depth, suggesting that the gut microbiota data is sufficient, and the sequencing results are reasonable, and can reflect the most majority of microbial information in the sample. As shown in Figures 3C–F, microbial diversity analysis demonstrated statistically significant elevations in α-diversity indices for the GPS-1 intervention group compared to control group (*P* < 0.05, *P* < 0.01), including community richness estimators (ACE and Chao1 indices) and diversity metrics (Simpson and Shannon indices). In addition, the overall differences between the microbial community structures of different groups were assessed by principal component analysis (PCA, Figure 3G) and principal coordinate analysis (PCoA, Figure 3H). The results showed that the microbial communities of the GPS-1 group were significantly different and clearly separated from those of the control group, as indicated by both PCA and PCoA ordination analyses. These results suggest that GPS-1 can enhance the diversity and richness of the gut microbiota.

#### 3.4.2 Effects of GPS-1 on the composition and function of gut microbiota

**FIGURE 4:**
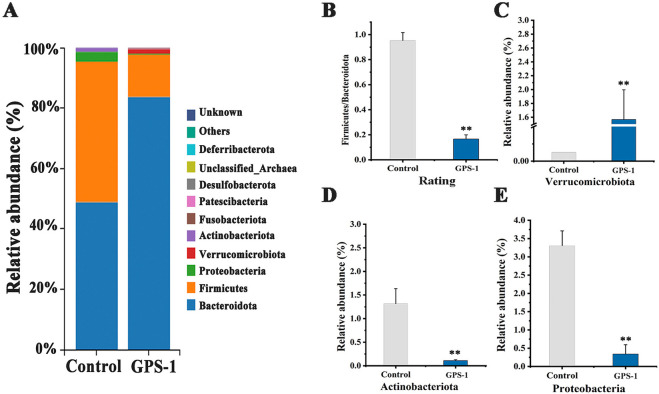
**(A)** Gut microbiota composition at the phylum level; **(B)** Ratio of firmicutes to bacteroidetes; **(C–E)** Changes in characteristic bacteria at the phylum level.

As shown in Figure 4, at the phylum level, *Firmicutes*, *Bacteroidota*, and *Verrucomicrobia* were the dominant bacteria in the control group, with content of 46.46%, 48.85%, and 1.31%, respectively (Figure 4A). Microbiota profiling showed distinct changes in microbial structure in the GPS-1 intervention group compared to the control group, demonstrating increased relative abundances of *Patescibacteria* and *Verrucomicrobia* (Figure 4C) (*P* < 0.01), while exhibiting concurrent reductions in *Desulfobacterota*, *Firmicutes*, *Actinobacterito* (Figure 4D), and *Proteobacteria* (Figure 4E) (*P* < 0.01). Particularly noteworthy was the significant decrease in the Firmicutes/Bacteroidetes (F/B) ratio (Figure 4B, *P* < 0.01), suggesting GPS-1-induced remodeling of gut microbial architecture.

**FIGURE 5:**
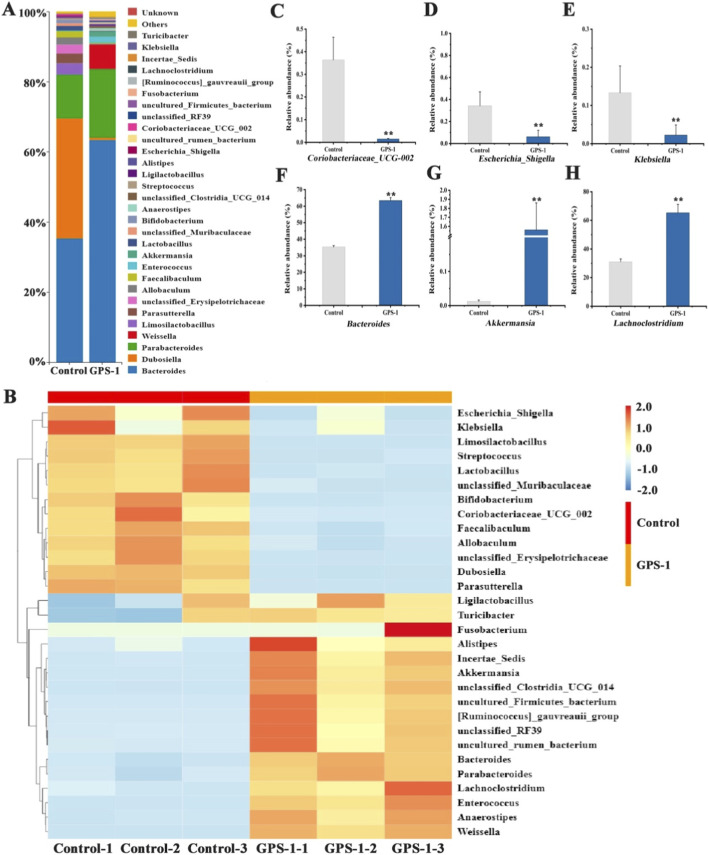
**(A)** Bar chart of gut microbiota composition changes at the genus level; **(B)** Heat maps of Gut microbiota composition changes at the genus level; **(C–H)** Changes in characteristic bacteria at the genus level. ^*^ *P* < 0.05, ^**^ *P* < 0.01 compared with the control group.

At the genus level, T2DM induced an increase in the abundance of potentially pathogenic bacteria such as *Coriobacteriaceae_UCG-002*, *Escherichia_Shigella*, *Klebsiella*, *Lactobacillus*, and *Parasutterella* (Figures 5A–E). However, GPS-1 decreased the abundance of *Coriobacteriaceae_UCG-002, Escherichia_Shigella*, and *Klebsiella*, while increasing the abundance of beneficial bacteria such as *Bacteroides*, *Akkermansia*, *Lachnoclostridium,* and others (Figures 5F–H). These results indicated that the effects of GPS-1 on lipid metabolism in type 2 diabetes mellitus may be mediated through its modulation of gut microbiota.

**FIGURE 6:**
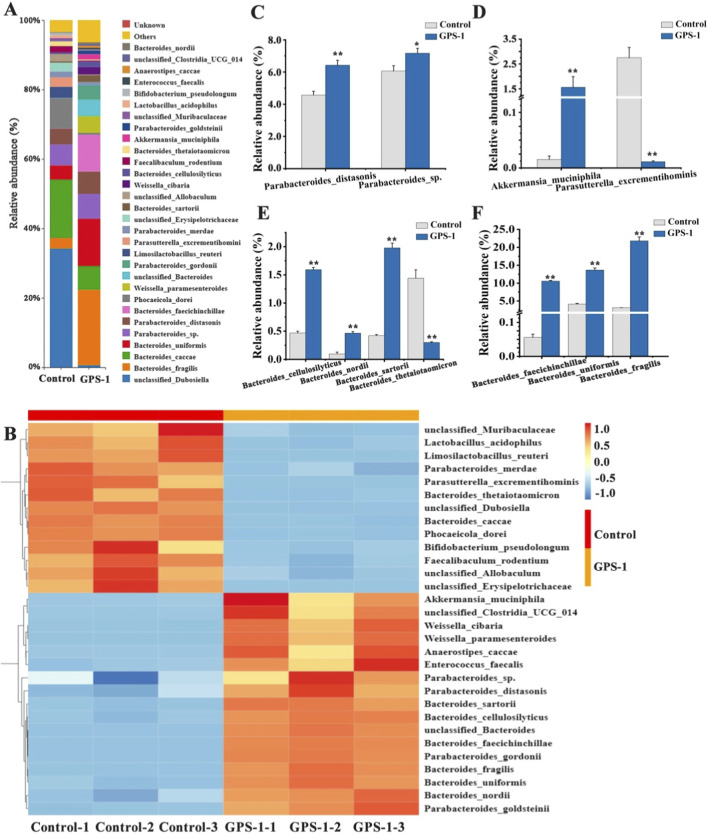
**(A)** Bar chart of gut microbiota composition changes at the species level; **(B)** Heat maps of gut microbiota composition changes at the species level; **(C–F)** Changes in characteristic bacteria at the species level.

Furthermore, GPS-1 treatment significantly modulated bacterial populations in T2DM rats, with notable reductions in potentially pathogenic taxa including *Parasutterella-excrementihominis*, unclassified*-Allobaculum*, and unclassified*-Erysipelotrichaceae* (Figures 6A–F). Conversely, the GPS-1 group exhibited significant enrichment of beneficial microbial species such as *Akkermansia-muciniphila*, unclassified*-Clostridia-UCG-014*, *Weissella-cibaria*, *Weissella-paramesenteroides*, *Anaerostipes-caccae*, along with multiple *Parabacteroides species (Parabacteroides-distasonis*, *Parabacteroides-gorsonii*, *Parabacteroides-goldsteinii*) and other beneficial bacteria were observed in GPS-1-treated group. Importantly, among the top 30 most abundant gut microbial species in the GPS-1 group, nine belonged to the genus *Bacteroides*, compared to the control group. This *Bacteroides* enrichment specifically included *B*. *cellulosilyticus*, *B*. *nordii*, *B*. *sartorii*, *B. faecichinchilla*, *B*. *uniformis*, and *B. fragilis*.

**FIGURE 7:**
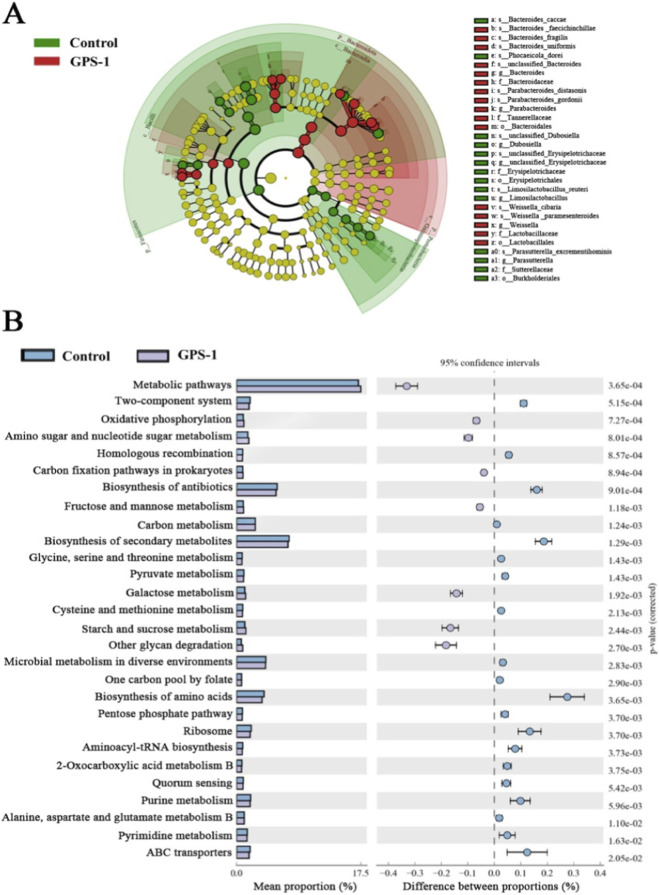
LEfSe analysis and functional predictions. **(A)** LEfSe taxonomic cladogram; **(B)** functional predictions of gut microbiota based on KEGG pathways.

LEfSe analysis confirmed that GPS-1 specifically enriches the genus *Bacteroides* (Figure 7A). Correspondingly, KEGG functional prediction revealed that GPS-1 intervention significantly enhanced microbial metabolic activities (Figure 7B). Key pathways including two-component system, oxidative phosphorylation, and carbon metabolism were markedly activated in the GPS-1 group. Furthermore, pathways related to polysaccharide degradation and utilization, such as amino sugar and nucleotide sugar metabolism, were also enriched. Conversely, the gut microbiota in the Control group exhibited significant enrichment in pathways like pyrimidine metabolism and ABC transporters. Based on the results of LEfSe and KEGG analyses, the aforementioned findings preliminarily elucidate the mechanism by which GPS-1 may alleviate metabolic disorders in type 2 diabetes through the modulation of gut microbiota and related metabolic pathways.

### 3.5 Effect of GPS-1 on SCFAs

This study further evaluates the capacity of GPS-1 to stimulate SCFAs production. As summarized in Table 4, GPS-1 treatment significantly increased the levels of acetic acid, propionic acid, n-butyric acid, i-butyric acid, n-valeric acid, i-valeric acid, and n-caproic acid compared to the control group. In addition, the total SCFA content exhibited a time-dependent increase during fermentation. After 48 h of fermentation, the total SCFA content in the GPS-1 group was 10.57-fold higher than its baseline level and 4.16-fold higher than that in the control group at the same time point (*P* < 0.01). Furthermore, after 48 h of fermentation, the GPS-1 treated group showed significantly higher levels of all seven quantified SCFAs than the control group, particularly acetate and propionate, which were 4.48-fold and 2.07-fold higher, respectively (Table 4). Spearman correlation analysis further confirmed significant associations between specific bacterial genera and SCFA production (Supplementary Figure S2). Acetate showed a significant positive correlation with *Akkermansia* and *Bacteroides*; propionate correlated most strongly with *Bacteroides*; and butyrate was positively correlated with Firmicutes-associated genera such as *Lachnoclostridium* and *Anaerostipes*. Additionally, certain potential pathogens like *Escherichia_Shigella* exhibited negative correlations with multiple SCFAs. These results further suggest that GPS-1 may improve the metabolic status of T2DM rats by correcting gut microbiota dysbiosis and promoting the production of beneficial metabolites, SCFAs.

**TABLE 4:** Changes in short-chain fatty acid content during fermentation (mmol/L, Mean ± SD, n = 3).

Groups	Time(h)	Acetic acid	Propionic acid	n-Butyric acid	i-Butyric acid	n-Valeric acid	i-Valeric acid	n-Caproic acid	Total
Control	0	0.633 ± 0.032	0.036 ± 0.001	0.055 ± 0.001	0.014 ± 0.001	0.022 ± 0.001	0.007 ± 0.003	0.004 ± 0.001	0.771 ± 0.096
	12	0.774 ± 0.342	0.062 ± 0.029	0.053 ± 0.001	0.033 ± 0.006^*^	0.007 ± 0.002	0.016 ± 0.003	0.013 ± 0.002	0.958 ± 0.074
	24	1.552 ± 0.103^**^	0.055 ± 0.036	0.106 ± 0.004^*^	0.024 ± 0.022	0.004 ± 0.004	0.016 ± 0.003	0.007 ± 0.002	1.764 ± 0.083^**^
	36	1.730 ± 0.000^**^	0.065 ± 0.005^*^	0.091 ± 0.042^*^	0.031 ± 0.001^*^	0.007 ± 0.003	0.018 ± 0.002^*^	0.011 ± 0.004^*^	1.953 ± 0.203^**^
	48	2.212 ± 0.244^**^	0.194 ± 0.005^**^	0.217 ± 0.010^**^	0.048 ± 0.013^*^	0.004 ± 0.001	0.027 ± 0.002^**^	0.008 ± 0.002	2.710 ± 0.033^**^
GPS-1	0	0.642 ± 0.097	0.152 ± 0.004	0.140 ± 0.006	0.022 ± 0.003	0.002 ± 0.000	0.012 ± 0.001	0.005 ± 0.001	0.975 ± 0.116^##^
	12	1.677 ± 0.214^*#^	0.121 ± 0.041^#^	0.178 ± 0.006^#^	0.042 ± 0.005^*^	0.004 ± 0.003	0.021 ± 0.005	0.010 ± 0.002	2.053 ± 0.082^*##^
	24	4.828 ± 0.146^**##^	0.109 ± 0.047^#^	0.442 ± 0.005^**##^	0.043 ± 0.004^*#^	0.007 ± 0.003	0.026 ± 0.006	0.014 ± 0.006^*^	5.469 ± 0.542^*##^
	36	7.586 ± 1.000^**##^	0.287 ± 0.053^*##^	0.551 ± 0.012^**##^	0.047 ± 0.001^*^	0.006 ± 0.001	0.022 ± 0.000	0.010 ± 0.002	8.509 ± 0.490^**##^
	48	9.912 ± 0.526^**##^	0.402 ± 0.034^**#^	0.827 ± 0.009^**##^	0.067 ± 0.013^**^	0.007 ± 0.001	0.039 ± 0.002^*^	0.015 ± 0.001^*^	11.269 ± 0.459^**##^

^*^ *P* < 0.05, ^**^ *P* < 0.01 vs. 0-h fermentation, ^#^ *P* < 0.05, ^##^ *P* < 0.01 vs. control group at the same fermentation time point for SCFAs, content.

### 3.6 Cytotoxicity of GPS-1 and its fermentate

**FIGURE 8:**
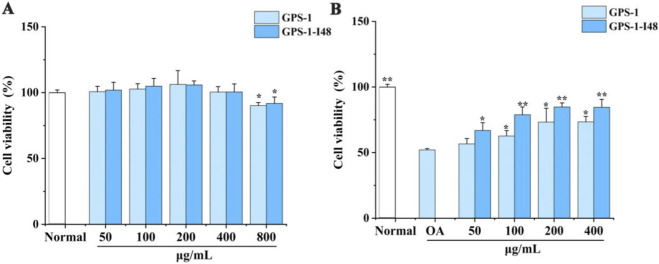
**(A)** Cell viability of GPS-1 and GPS-1-I48 in HepG2 cells. ^*^ *P* < 0.05, ^**^ *P* < 0.01 compared with the Normal group; **(B)** Cell viability of GPS-1 and GPS-1-I48 on OA-induced HepG2 cells. ^*^ *P* < 0.05, ^**^ *P* < 0.01 compared with the OA-treated group.

Based on our previous demonstration that GPS-1 alleviates lipid accumulation in liver of T2DM rats via gut microbiota modulation, this study employed an *in vitro* digestion and fermentation model to prepare the GPS-1 fermentation product GPS-1-I48, and subsequently evaluated for their improvement against OA-induced lipid accumulation in HepG2 cells. The cytotoxicity of GPS-1 and GPS-1-I48 was first assessed by CCK-8 assay. As shown in Figure 8A, no significant cytotoxicity was observed at concentration up to 400 μg/mL compared with the normal group. At 400 μg/mL, GPS-1 and GPS-1-I48 exhibited comparable viability (95.49% ± 7.75% and 98.88% ± 6.09%) to the normal group, whereas 800 μg/mL induced significant cytotoxicity (viability <90%, *P* < 0.05). Subsequent experiments were therefore conducted within the range of 100–400 μg/mL.

### 3.7 Effect of GPS-1 and its fermentate on OA-induced HepG2 cell viability

The results demonstrate that OA-treatment significantly reduced HepG2 cell viability by 48.1%, whereas both GPS-1 and its fermentate GPS-1-I48 effectively counteracted this OA-induced decline. At equivalent concentrations, GPS-1-I48 exhibited superior protective activity compared to GPS-1 (Figure 8B).

### 3.8 Effect of GPS-1 and its fermentate on lipogenesis in OA-induced HepG2 cell

**FIGURE 9:**
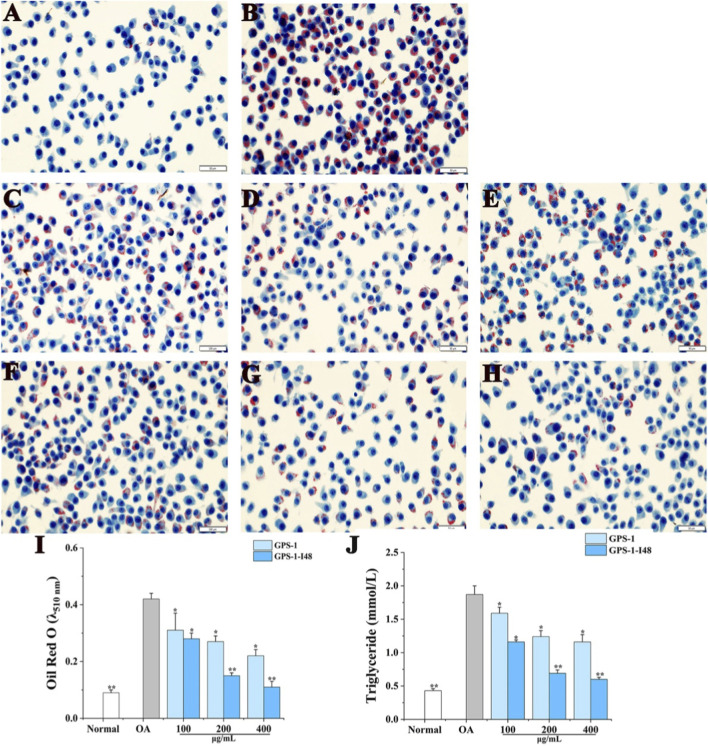
Inhibitory effect of GPS-1 and GPS-1-I48 on OA-induced lipid accumulation in HepG2 cells. **(A–H)** Intracellular lipid accumulation was visualized by Oil Red O staining using an inverted microscpoe (×400 magnification); **(I)** Lipid accumulation was quantified with a microplate reader at 510 nm; **(J)** Intracellular triglyceridein levels. ^*^ *P* < 0.05, ^**^ *P* < 0.01 compared with the OA-treated group.

As shown in Figures 9A–I, cells in normal group had clear edges, intact nuclear membranes, and only a small number of cells showed red lipid droplets. Compared with the normal group, the OA treatment caused a large number of lipid accumulation in HepG2 cell, as well as the TG level was markedly increased indicating the successful establishment of cell model with lipid metabolism disorders (Figure 9J). Both GPS-1 and its fermented product (GPS-1-I48) significantly attenuated lipid accumulation and reduced TG levels compared with the OA-treated group, with the efficacy following the order: GPS-1-I48 > GPS-1.

### 3.9 Effect of GPS-1 and its fermentate on the antioxidant system in OA-induced HepG2 cell

**FIGURE 10:**
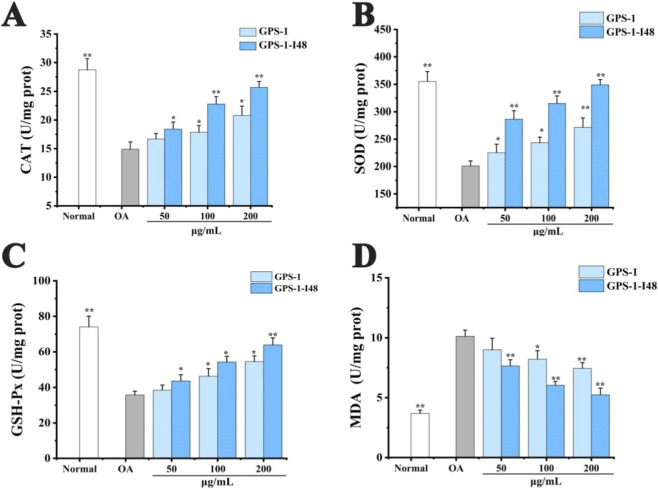
Effects of GPS-1 and GPS-1-I48 on antioxidant enzymes and MDA levels in oleic acid-induced HepG2 cells at different dose groups (Mean ± SD): **(A)** CAT; **(B)** SOD; **(C)** GSH-Px; **(D)** MDA. ^*^ *P* < 0.05, ^**^ *P* < 0.01 compared with the OA-treated group.

As shown in Figure 10, the OA-treated group exhibited 2.75-fold higher MDA level coupled with 43.41%, 48.14%, and 51.67% decrease in SOD, CAT, GSH activities compared to that of the normal group, indicating successful oxidative stress conditions. Notably, GPS-1-I48 showed the stronger antioxidant capacity compared to GPS-1 at equivalent concentrations. Compared with the OA-treated group, GPS-1-I48 exhibited significant dose-dependent antioxidant activity from a dosage of 50 μg/kg, with the optimal effect observed at 200 μg/kg. At this dosage, GPS-1-I48 administration resulted in a 33.20% inhibition of MDA production, while upregulating the activities of SOD by 46.15%, CAT by 49.09%, and GSH-Px by 56.87%, respectively. These findings indicated that GPS-1-I48 could mitigate oxidative stress-induced cellular damage through enhancing the activity of antioxidant enzymes and direct scavenging of lipid peroxides.

## 4 Discussion

Accumulating evidence suggests that the microbial fermentation of plant-derived polysaccharides in the gut may constitute a fundamental mechanism underlying their bioactivities, a process that has recently garnered significant scientific attention. As indigestible dietary components in hosts, the metabolism of pectin is critically dependent on gut microbiota-mediated degradation and utilization. Notably, pectin exhibits bidirectional interactions with gut microbiota: it not only functions as a prebiotic to optimize microbial community structure, but also undergoes microbial fermentation to generate bioactive metabolites such as SCFAs, thereby conferring health benefits. Our previous study demonstrated that red ginseng pectin GPS-1 could ameliorate lipid metabolism disorders in T2DM rats through gut microbiota modulation, yet its precise digestive and fermentative characteristics remain elusive. Utilizing an *in vitro* digestion-fermentation model, the current investigation revealed that GPS-1 not only restructured the fecal microbiota composition in T2DM rats, particularly enhancing *Bacteroides* proliferation, but was also effectively metabolized by gut microbiota to promote SCFAs production. Remarkably, the fermented GPS-1 derivatives exhibited substantially enhanced therapeutic efficacy in alleviating oleic acid-induced lipid dysregulation in HepG2 cells.

The digestion of plant polysaccharides generally refers to the process occurring in simulated animal digestive tract environments (including oral cavity, stomach, and small intestine). Characteristic physicochemical parameters of polysaccharides, such as changes in *Mw* distribution, monosaccharide composition, and C_R_ levels, serve as critical indicators reflecting their digestive properties (Karppinen et al., 2020). In this study, no significant changes were observed in total sugar content, C_R_ content, uronic acid content, or *Mw* of GPS-1 throughout the oral, gastric, and small intestinal digestion phases, suggesting partial degradation of non-amyloid polysaccharides under acid gastric conditions with digestive enzymes. Furthermore, the monosaccharide composition of GPS-1 remained stable throughout digestion, except for a 7.88% decrease in Ara content specially in the small intestine phase. No significant alterations were found in either the variety or proportions of other constituent monosaccharides. This phenomenon is likely attributable to the relative susceptibility of glycosidic bonds formed by Ara residues under alkaline conditions or specific enzymatic actions characteristic of the small intestinal environment. Collectively, the integrated results from the aforementioned analyses and FT-IR spectroscopic data demonstrate that only a minor portion of GPS-1 underwent digestion throughout the oral-gastric-intestinal digestion process. Previously studies suggest that the polysaccharide digestion properties are influenced by *Mw*, monosaccharide composition, glycosidic linkage patterns and conformation (Payling et al., 2020). In addition, most non-starch polysaccharides resist dalivary digestion but can be limited hydrolyzed in acidic gastric environments with digestive enzymes (Payling et al., 2020). Furthermore, high *Mw* polysaccharide often exhibit gastrointestinal stability (Hamaker and Tuncil, 2014). Our data demonstrate that GPS-1 exhibits structural resistance to *in vitro* salivary-gastrointestinal digestion under acidic environment. This stability likely arises from its high molecular weight and specific glycosidic bonding pattern, which impede enzymatic and acidic degradation in both saliva and gastric environments.

In contrast, during the fermentation phase, over 70% of GPS-1 was consumed. A marked increase in C_R_ content (from 0.0793 mg/mL to 0.1387 mg/mL) was observed, especially in the end stage of fermentation (12 h–24 h). The cleavage of glycosidic bonds in polysaccharides typically correlates with increased C_R_ levels, and the changes in C_R_ content reflect polysaccharide utilization efficiency, suggesting the metabolic accessibility of polysaccharide by gut microbiota (Karppinen et al., 2020). Furthermore, the C_R_ levels in fermentation phase displayed a trend of first increase and then decrease. This dynamic suggests that metabolically active gut microbiota hydrolyzed GPS-1 glycosidic bonds during early fermentation (0–24 h), generating reducing sugars. Subsequently, some of these sugars were preferentially utilized by the microbial community, resulting in C_R_ depletion during later stages (36–48 h) (Gao et al., 2018). In addition, the structural variations of polysaccharides, particularly *Mw* distribution, monosaccharide composition, and characteristic functional groups constitute critical indicators for assessing their behaviors during fermentation (Ma et al., 2021). A reduction in *Mw* of GPS-1 suggested that it was broken down with prolonged fermentation, and these results were consistent with the changes in total sugar content, C_R_ levels, and uronic acid concentrations throughout the *in vitro* fermentation processes. The degradation of natural polysaccharides during *in vitro* digestion and fermentation also manifests as changes in monosaccharide composition. In this work, no significant change in monosaccharide composition was observed from oral to gastric stages, but the monosaccharide molar ratio profile of GPS-1 underwent significant alterations during the fecal fermentation process. Notably, the main structural components of pectin, including GalA and Ara, exhibited a progressive decline with prolonged fermentation. Conversely, Rha and minor constituents Glc and Fuc in GPS-1 displayed a gradual accumulation. These observations demonstrate that both the side chain and backbone of GPS-1 were degraded by the fecal microbiota of T2DM rats. Specifically, after 48 h of fermentation, the contents of Ara were reduced by over 80%. The pectic polysaccharide extract from okra showed that after 6 h of fermentation, the pectin side chains became more susceptible to degradation and utilization by the gut microbiota. This phenomenon may also explain the significant decrease in Ara content observed in GPS-1 in this study (Wu et al., 2021c).

The enzymatic degradation of pectin mediated by gut microbiota-derived CAZymes is invariably accompanied by structural modifications of some functional groups, particularly carboxyl groups (C=O) and methyl ester moieties (-OCH_3_). FT-IR usually serves as a useful analytical tool to monitor these dynamic changes, specifically through characteristic absorption bands at 1730 cm^-1^ (C=O stretching vibrations of methyl-esterified COO^−^) and 1,620 cm^-1^ (C=O stretching vibrations of free COO^−^), which are critical biomarkers for tracking pectin de-esterification and depolymerization processes. The study by Wu et al. also confirmed that fermentation process reduces the degree of methylation in pectic polysaccharides, likely due to pectin esterases hydrolysis of methyl ester groups, leading to de-esterification (Wu et al., 2021c).

This investigation yielded consistent findings with previous observations. Though the digestive products exhibited analogous FT-IR spectral patterns, confirming structural preservation of the polysaccharide backbone, the esterified carboxyl C=O stretching vibration at 1730 cm^-1^ in GPS-1 demonstrated time-dependent attenuation during fermentation (0–48 h), which directly corresponds to the gradual de-esterification of GPS-1. However, other studies have observed that polysaccharide functional groups remain unaltered during fermentation process, which may be related to their specific structural characteristics (Ma et al., 2021). This collective evidence further substantiates that GPS-1 was predominantly degraded by the fecal microbiota of T2DM rats during the fermentation phase. Moreover, the compositional changes revealed distinct molar ratio of the sugar residues in the fermentation products (GPS-1 s), confirming structural change during the *in vitro* fermentation process.

A bidirectional regulatory mechanism operates between gut microbiota and polysaccharides: the microbiota metabolizes dietary polysaccharides via glycolytic pathways, while polysaccharides act as prebiotics to shape microbial community structure and regulate metabolite biosynthesis (Wu. et al., 2021a). Since the human body lacks the enzymes required to directly hydrolyze non-starch polysaccharides, most of these polysaccharides are mainly degraded and utilized by gut microbiota. The resulting enzymatic products can be utilized by bacteria, thereby influencing the composition of the microbial community (Zhu et al., 2020). Our investigation first focus on the modulatory effects of GPS-1 on fecal microbiota in T2DM rats during fermentation processes. Analytical results demonstrated GPS-1’s significant structural remodeling of microbial communities across phylum, genus, and species taxonomies. Particularly, GPS-1 effectively attenuated the elevation of the F/B ratio. Studies have confirmed that the F/B ratio of gut microbiota is significantly elevated in obese animal models (Lai et al., 2024). This aligns with documented mechanisms wherein dietary fibers like pectin ameliorate metabolic disorders through F/B ratio normalization (Wu et al., 2021c). Crucially, our intervention achieved 82.50% reduction in F/B ratio compared to control group. Notably, specific members within the Desulfobacterota and Proteobacteria phyla are recognized as opportunistic pathobionts in intestinal homeostasis. Accumulating evidence indicates that dietary plant polysaccharides can restructure gut microbial ecosystems by selectively inhibiting the overgrowth of these detrimental taxa, thereby improving gut health (Wu et al., 2017; Gu et al., 2025). Our results revealed that GPS-1 significantly reduced the relative abundance of Proteobacteria compared to diabetic controls (*P* < 0. 01), Consisting with the results reported in the literature. Furthermore, GPS-1 selectively reduced the relative abundance of diabetes-associated opportunistic pathogens including *Escherichia_Shigella*, *Parasutterella*, *Coriobacteriaceae_UCG-002*, and *Allobaculum*, while concomitantly enriching beneficial genera such as *Bacteroides*, *Alistipes*, *Akkermansia*, and *Lachnoclostridium* at the genus level (*P* < 0.05 vs. control). This microbial shift aligns with established pathogenic patterns in T2DM, where elevated abundances of *Escherichia_Shigella* and *Coriobacteriaceae_UCG-002* both positively correlated with lipid levels and obesity (Ren et al., 2025). Pectin exerts lipid-modulating effects partly by enriching *Akkermansia*, while synergistically enhancing *Bifidobacterium*, *Bacteroides*, and *Prevotella* and *Saccharibacteria_genera_incertae_sedis* that collectively regulate hepatic lipid metabolism pathways (Ren et al., 2023). Additionally, the study findings confirmed that the increased abundance of these microbial species exhibited a strong positive correlation with elevated SCFA production. This suggests that GPS-1 may regulate SCFA output through potential targeting of specific SCFA-producing gut microbiota, such as enriching *Akkermansia* and *Bacteroides* species.

The *in vitro* digestion-fermentation model, which integrates multiple physiological barriers including oral digestion, gastric acid hydrolysis, intestinal enzymatic degradation, and gut microbial fermentation, provides a crucial research platform for elucidating the dynamic evolution mechanisms of polysaccharide bioactivity. In the current study, GPS-1 was degraded by fecal microbiota of T2DM rats, producing various fermentation products such as monosaccharides, oligosaccharides, and SCFAs, etc. Post-fermentation GPS-1 in T2DM rats demonstrated improved efficacy in suppressing oleic acid-induced lipid accumulation and significantly alleviating oxidative damage. This functional enhancement may correlate with structural modifications and microbial metabolic synergy during digestion-fermentation processes, including *Mw* reduction, altered monosaccharide composition, and functional group remodeling. Specifically, the breakdown of high-molecular-weight polysaccharides into low-molecular-weight fragments or oligosaccharides through microbial enzymes, such as pectinases, likely increased their bioavailability and exposed functional groups (e.g., uronic acids) that directly interact with cellular receptors involved in lipid metabolism (Dilip et al., 2019; Gilbert et al., 2018). This hypothesis aligns with previous studies showing that microbiota-derived metabolites potentiate the bioactivity of dietary polysaccharides. Studies demonstrate that the digestion-fermentation process can significantly modify polysaccharide biological activities through structural alterations. Gu et al. employed this model to investigate the degradation dynamics and immunomodulatory mechanisms of *Lycium barbarum* polysaccharide (LBPS). Their findings revealed that LBPS underwent progressive *Mw* reduction from 1.236 × 10^5^ Da to 3 × 10^4^ Da through sequential treatment with salivary α-amylase, pepsin, and pancreatic enzymes, exposing cryptic β-1,3-glucan active fragments. These digested products were further converted by fecal microbiota into oligosaccharide fragments (<5 × 10^3^ Da), which activated macrophage TLR4 receptors to initiate NF-κB signaling pathways, ultimately resulting in a 52% significant increase in IL-6 secretion compared to undigested controls, confirming that the multi-stage digestion-fermentation process enhances polysaccharide immunogenicity (Gu et al., 2025). Furthermore, Xu et al. discovered that digested-fermented products of ginseng non-starch glucan (DGPN-F48) exhibited enhanced activation of the TLR4/Myd88/NF-κB signaling pathway, demonstrating superior macrophage polarization induction capacity (Xu et al., 2025). It should be noted that this study builds upon prior experimental findings to further investigate the interaction between GPS-1 and the gut microbiota of T2DM rats using *in vitro* fermentation, and subsequently explores the impact of microbial fermentation on its bioactivity. Consequently, all experiments in this study utilized fecal microbiota from T2DM rats as the inoculum, and the results may not be directly generalizable to the gut microbial ecosystem of healthy individuals. Existing studies have demonstrated systematic differences between the gut microbiota of healthy and T2DM individuals in terms of diversity, the abundance of key bacterial genera, and functional profiles (Wu et al., 2021a; Ma et al., 2021). Therefore, the fermentation characteristics of GPS-1, its regulatory effects on microbiota structure, and its patterns of SCFA production may differ in a healthy microbial environment. Future research should establish control experimental groups inoculated with fecal samples from healthy individuals or animals. Through direct comparison, such studies could systematically clarify both the commonalities and specificities of the interactions between GPS-1 and gut microbiota under different health statuses. This will contribute to a more comprehensive evaluation of the potential applicability and efficacy of GPS-1 as a prebiotic functional ingredient.

## 5 Conclusion

In conclusion, our findings demonstrate that GPS-1 exhibits remarkable resistance to degradation during simulated saliva-gastrointestinal digestion. Subsequent *in vitro* fecal fermentation by gut microbiota triggered substantial structural modifications, including depolymerization of *Mw*, altered monosaccharide composition, and decreased esterification degree. These transformations were accompanied by characteristic fermentation parameters (pH decline and OD600 elevation). Notably, GPS-1 fermentation significantly modulated gut microbial composition, as evidenced by the remarkable enrichment of beneficial bacteria such as *Bacteroides* and *Akkermansia*, concurrently with the reduction of harmful bacteria. This microbial remodeling was functionally linked to a significant increase in SCFAs production, particularly propionate and acetate. Most importantly, the fermented product GPS-1-I48, derived from this microbiota-driven process, exhibited superior efficacy compared to native GPS-1 in alleviating OA-induced lipid accumulation and oxidative damage in HepG2 cells. Collectively, these results establish that the hepatoprotective effect of GPS-1 is dependent on gut microbial fermentation. Our study not only elucidates the causal relationship between GPS-1 fermentation and its bioactivity but also positions it as a promising prebiotic candidate for preventing lipid metabolism disorders through gut microbiota-mediated mechanisms. However, as the fermentation product GPS-1-I48 likely constitutes a heterogeneous mixture comprising residual polysaccharides, oligosaccharides, and SCFAs. Thus, the current experimental system cannot attribute the observed efficacy to any single component. In further studies, we will isolate individual components and validate their respective bioactivities to better elucidate their specific contributions and potential synergies, thereby advancing the understanding of the bioactive basis of GPS-1-I48.

## Data Availability

The data presented in the study are deposited in the NCBI (SRA) repository, accession number PRJNA1419354. Available at: https://www.ncbi.nlm.nih.gov/sra/PRJNA1419354/PRJNA1419354.
