# Supplementary material for: In vitro digestion and fermentation of ginseng pectic polysaccharide GPS-1 and attenuation of its product on oleic acid-induced oxidative stress in HepG2 cells
Source: Front Pharmacol. 2026 Feb 18;17:1758770. doi: 10.3389/fphar.2026.1758770 (PMC12957194; doi:10.3389/fphar.2026.1758770)
Supplement: Supplementary file 1 [file Supplementaryfile1.docx]

**Supplementary material**

**
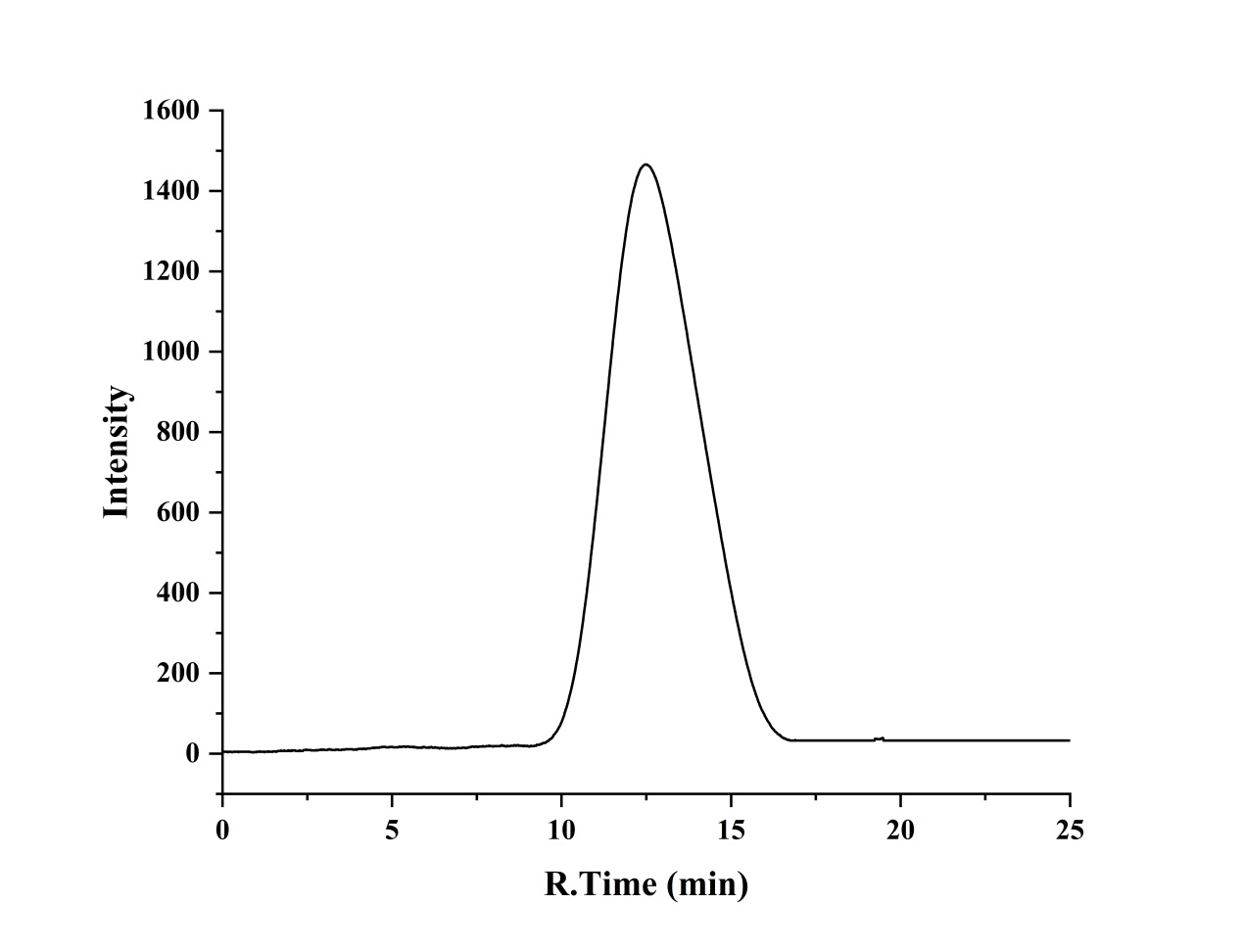
**

**Figure S1**  HPGPC spectrum of GPS-1.


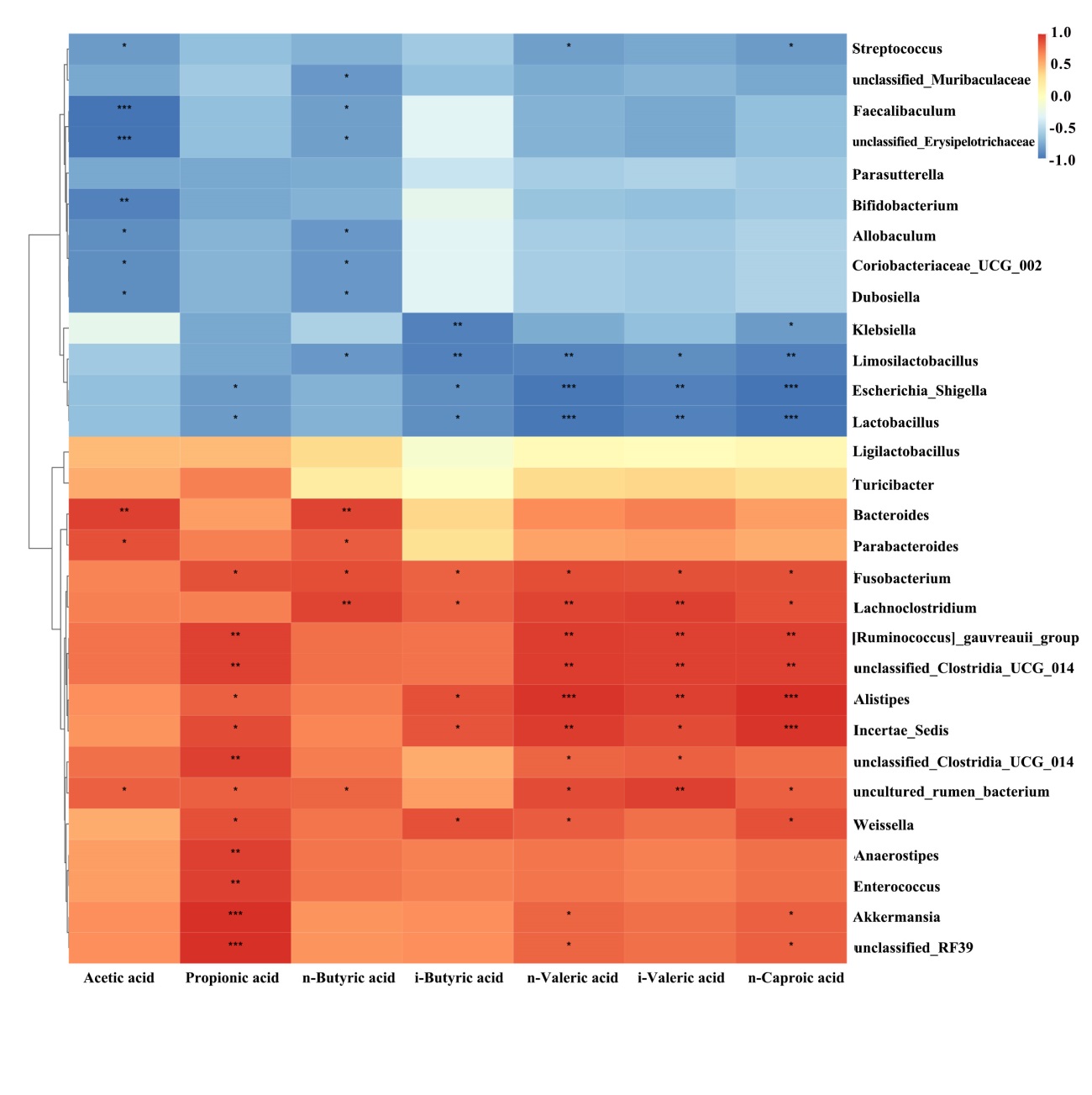


**Figure S2** Spearman correlation analysis between gut microbiota and SCFAs
